# A microfluidic live cell assay to study anthrax toxin induced cell lethality assisted by conditioned medium

**DOI:** 10.1038/srep08651

**Published:** 2015-03-03

**Authors:** Jie Shen, Changzu Cai, Zhilong Yu, Yuhong Pang, Ying Zhou, Lili Qian, Wensheng Wei, Yanyi Huang

**Affiliations:** 1 Biodynamic Optical Imaging Center (BIOPIC), Peking University, Beijing, 100871, China; 2 College of Engineering, Peking University, Beijing, 100871, China; 3 School of Life Sciences, Peking University, Beijing, 100871, China

## Abstract

It is technically challenging to investigate the function of secreted protein in real time by supply of conditioned medium that contains secreted protein of interest. The internalization of anthrax toxin is facilitated by a secreted protein Dickkopf-1 (DKK1) and its receptor, and eventually leads to cell lethality. To monitor the dynamic interplay between these components in live cells, we use an integrated microfluidic device to perform the cell viability assays with real-time controlled culture microenvironment in parallel. Conditioned medium, which contains the secreted proteins from specific cell lines, can be continuously pumped towards the cells that exposed to toxin. The exogenous DKK1 secreted from distant cells is able to rescue the sensitivity to toxin for those DKK1-knocked-down cells. This high-throughput assay allows us to precisely quantify the dynamic interaction between key components that cause cell death, and provide independent evidence of the function of DKK1 in the complex process of anthrax toxin internalization.

Anthrax, a lethal infectious disease for human and other animals, is caused by anthrax toxin that is secreted from Bacillus anthracis^1^,^2^. Anthrax toxin consists of three proteins; protective antigen (PA) and two toxic factors: lethal factor (LF) and edema factor (EF)^3^,^4^. None of these three proteins is toxic when it exists alone. However, PA can form toxic complex with LF or EF, and subsequently enters the target cell cytoplasm through anthrax toxin receptor-mediated endocytosis and finally causes the cell death^5^,^6^,^7^.

It is still challenging to identify additional key components that are related to the internalization and lethality of anthrax toxin. Further investigation of the internalization mechanism of anthrax toxin will not only deciphers the missing components in the pathological processes, but facilitates the discovery of therapeutic targets. It has been reported that low-density lipoprotein receptor-related protein 6 (LRP6) can mediate the internalization and lethality of anthrax toxin^8^. LRP6 is also a receptor for a secreted protein Dickkopf-1 (DKK1)^9^,^10^,^11^. In Wnt signaling pathway, DKK1 can form ternary complex with LRP6 and Kremen2, another membrane protein, for internalization^12^,^13^. We have recently reported that DKK1 plays an important role in the internalization and lethality of anthrax toxin through the ternary structure of LRP6-DKK1-Kremen2^14^. However, it is still technically challenging to investigate the function of secreted protein in real time without toilsome protein production and purification processes. One alternative approach is to use conditioned medium that contains secreted protein of interest. However, it is technically difficult to perform reproducible experiments with cells cultivated with conditioned medium, which is typically collected from other cell culture^15^,^16^. Moreover, medium acquisition and storage are often time-consuming and labor-intensive, making large-scale or multiplex experiments difficult to perform.

Microfluidics becomes an ideal technology to reduce the reaction volumes for highly parallel assays^17^,^18^,^19^,^20^,^21^. Recent advances in microfluidics have greatly improved the controllability and precision, enabling the dynamic and programmable microenvironment control of cell culture, especially for small number of cells^22^,^23^,^24^,^25^. Microfluidic devices have been applied to study the cell responses to secreted factors in the culture medium through two major ways: 1) create a chemical gradient, stable or dynamic, of soluble molecules by micro-patterns or well-regulated flows on-chip^26^,^27^,^28^, 2) constrain the physical distribution of different types of cells and control the direct or indirect interactions between cells^29^,^30^,^31^,^32^,^33^. Neither of these approaches is cost-effective to supply fresh conditioned medium, nor can they easily avoid the direct attachment between different types of cells.

Here we report a novel approach to accurately investigate how cells respond to the conditioned medium with specific soluble factors secreted from other live cells. We employed this approach to study the role of DKK1 in cell susceptibility to anthrax toxin. We identified a toxin-resistant LM/shRNA157B cell line, whose DKK1 expression level has been suppressed, and dynamically measured its viability under different culture conditions, with and without the exogenous DKK1 supplies (Figure 1a). These DKK1 proteins, freshly secreted from the wild-type LM cells that were cultured on-chip in a remote chamber, could be actively pumped into the culture chambers with DKK1-knocked-down cells. We found that exogenous DKK1 effectively rescued the cells' lost sensitivity to toxin, suggesting that DKK1 was likely serving as an activator in the internalization of anthrax toxin and consequently the cell death. Our high-throughput device could perform 4 groups of experiments, all with replicates, in 128 cell-culture chambers and 96 medium replacement chambers, providing an advanced technical solution to study the interaction between cells and soluble factors in the medium.

## Results and discussion

We fabricated the device using PDMS majorly because of its two intrinsic advantages: 1) PDMS is gas permeable, allowing air and CO_2_ to reach the medium in the closed culture chambers^34^; 2) PDMS is optically transparent with negligible background fluorescence, making the microscopic observation convenient and quantitative^35^. To better support the device, the chip was placed on a glass slide. To quantitatively study the biological function of proteins secreted from live cells, we carefully considered the layout and dynamic interactions between culture chambers that embedded in the device. Cells cultured in one chamber would not physically attach to the cells in other chambers while the pre-designed fluidic channels, with pneumatic valves^36^ to define the open/close status, linked the chambers.

**Figure 1:**
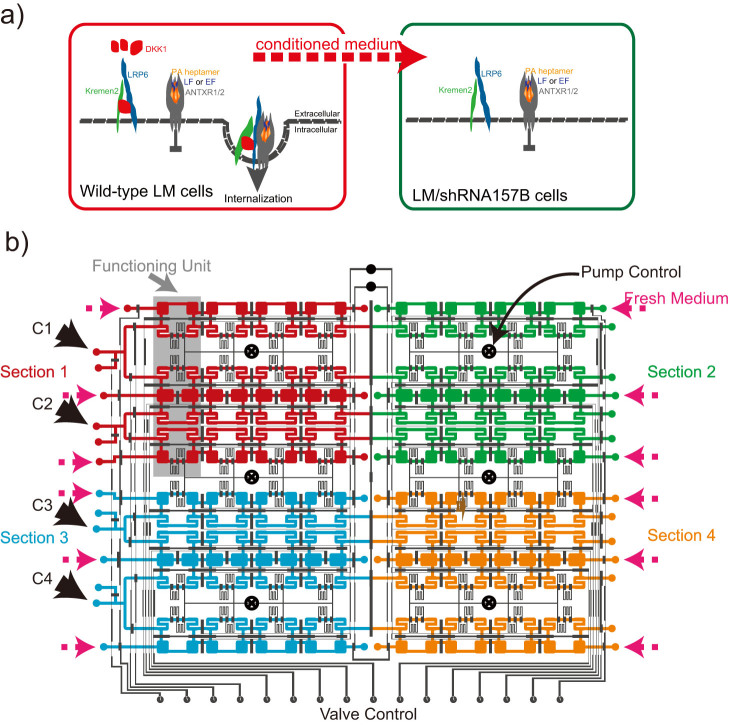
The schematic mechanism of anthrax toxin invasion process and the device layout. (a) Anthrax toxin (PA) induced cell lethality is assisted by DKK1, a secreted protein supplied by the conditioned medium collected from wild-type LM cells. (b) The layout of the whole device that contains 128 cell culture chambers and 96 medium-refresh chambers. The fluidic layer of the chip is illustrated as color-filled patterns, while the black lines represent the control lines and valves. The whole chip has 4 major inputs (C1 to C4) to load cells, and 12 inputs to introduce the culture medium. The chip can be divided into 4 sections and each section contains 4 functioning unit (highlighted with gray). 48 peristaltic pumps were actuated by 6 separate controls.

The layout of the whole device is shown in Figure 1b. The fluidic layer of the chip is illustrated as color-filled patterns. The whole chip has 4 major inputs (C1 to C4) to load cells into the culture chambers. Each input is responsible for 32 cell-culture chambers. There are also 12 inputs on the chip to introduce the culture medium. The control layer of the chip consists 22 control lines that can actuate 423 integrated pneumatic valves. Among them, 144 valves form 48 peristaltic pumps, which are divided into 6 groups.

**Figure 2:**
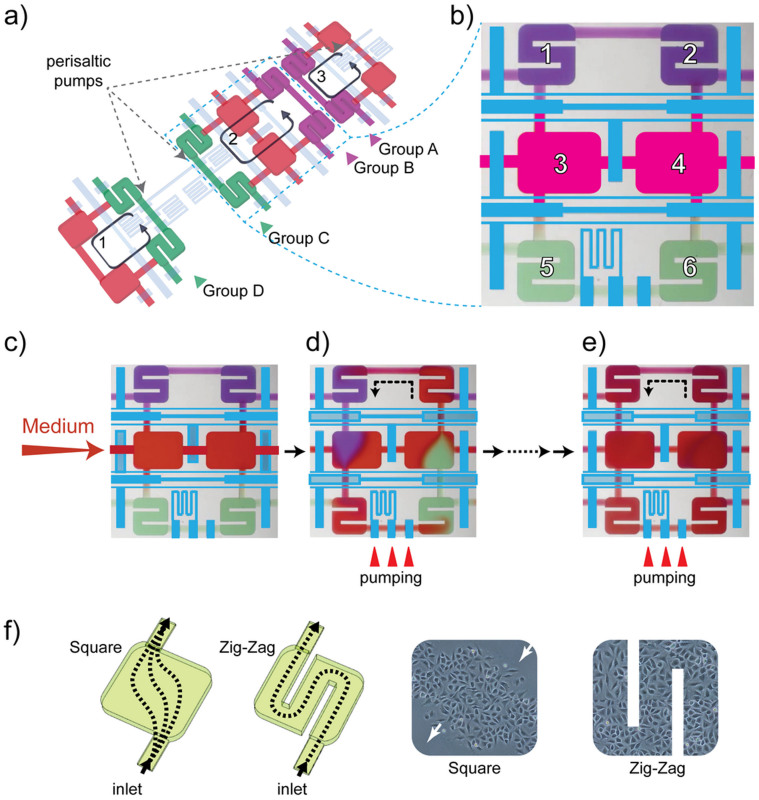
The key components of the device: fluidic exchange control between chambers. (a) The layout of a functioning unit with 14 chambers, 8 for cell culture (divided in 4 groups) and 6 for medium replacement, and 3 peristaltic pumps to drive 3 mixing routes between chambers (indicated with numbers). (b) A zoom-in view of the mixing route 2. During the experiment, one type of cells was cultured in chambers 1 and 2, the other in 5 and 6. (c) The central chambers 3 and 4 are used for medium replacement. (d) After the fresh medium is introduced, the peristaltic pump will mix the conditioned media of both cell lines, with dyes demonstrating different cultural environment. (e) The mixed culture medium will evenly distributed in all cell culture chambers after 10 min of mixing. (f) The flow characteristics of square and zigzag cell culture chambers, and the cell seeding results. White arrows indicated the stationary spots that cells rarely seed onto.

The chip can be viewed as the integration of 4 identical sections, and each section has 24 chambers for medium replacement and 32 chambers for cell culture, forming 4 identical replicating functioning units, one of which is highlighted in Figure 1b. The detailed structure of a functioning unit is illustrated in Figure 2a.

Every chip has 128 rectangular cell culture chambers. Each chamber is 1.20 mm × 1.30 mm × 0.05 mm, suitable to contain ca. 1,000 cells. The medium replacement chamber is 1.70 mm × 1.30 mm × 0.05 mm. 2 medium replacement chambers supply 4 cell culture chamber. Figure 2b show the key function of this device: fluidic exchange control between chambers. We load the top two chambers (1 and 2) with one type of cells and bottom two (5 and 6) with another type, leave the central two (3 and 4) free of cells. In this 2 × 3 configuration, every chamber is connected with the adjacent chamber with a short microfluidic channel, 300 μm wide and 25 μm high. These channels, with integrated pneumatic valves to open or close, are designed for medium exchange between chambers. One three-valve peristaltic pump^17^ has also been placed in this 6-chamber structure to drive the fluidic flow. We have found that medium refreshment is critical to keep cells healthy, and the refreshment may need to be more frequent than conventional culture due to the very limited volume in each culture chamber^37^. The chamber 3 and 4 were used to introduce fresh medium and replace the old one (Figure 2c). Then the replaced medium was mixed with the old one in the other 4 chambers by actuating the peristaltic pump (Figure 2d), and distributed uniformly in a few minutes among all the connected chambers (Figure 2e). In this 6-chamber configuration each operation will replace part of the medium, which brings sufficient nutrient, removes considerable amount of metabolic wastes, and still keep a big portion of the secreted soluble factors in the medium. Each functioning unit has a 6-chamber structure to mix the media from two types of cells, and two 4-chamber structures with similar designs serving as the control groups for both cell types (Figure 2a).

**Figure 3:**
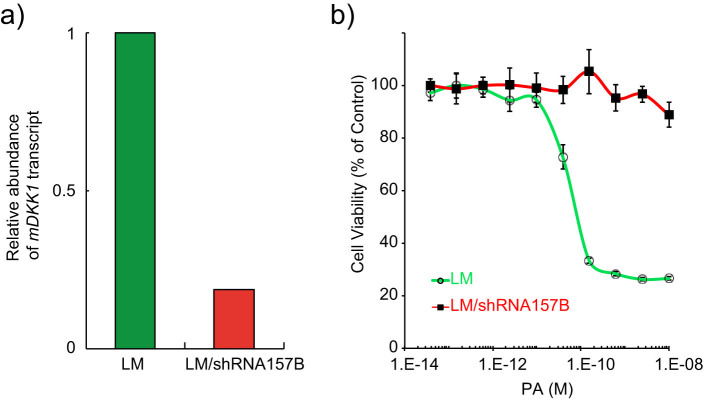
Effect of DKK1 down-regulation on LM cells. (a) DKK1 expression level measured by RT-PCR in LM cells and LM/shRNA157B cells, with shRNA stable expression. (b) The viabilities of LM cells and LM/shRNA157B cells against anthrax toxin PA/FP59 measured by MTT assay. Data are mean ± SD, n = 6.

The cell culture chambers were not just plain square boxes. We placed two bars in each chamber, shown in Figure 2f, and turned the chamber into a 3-section zigzag channel. This design was proved to be a simple yet effective solution which ensured that there would not be any stationary part in the chamber when medium flowed through, generating evenly seeded cells and uniform medium exchange in each chamber^35^. The flow characteristics of square and zigzag cell culture chambers, and the cell seeding results were shown in Figure 2f. White arrows indicated the stationary spots that cells rarely seed onto. The DKK1-knocked-down LM/shRNA157B cells and their corresponding control groups (wild type LM cells) were employed in our experiment. Every functioning unit contains 8 cell culture chambers, 4 loaded with wild type LM cells and the other 4 with LM/shRNA157B cells. We hence had 4 groups of experiments in parallel: LM cells (Group A), LM cells that shared secreted DKK1 with LM/shRNA157B (Group B), whose DKK1 expression level has been suppressed (Figure 3a), LM/shRNA157B cells with exogenous DKK1 freshly secreted from LM cells (Group C), and LM/shRNA157B cells without exogenous DKK1 (Group D). Each group has two technical replicates, and in total we have 4 biological replicates in each section. 12 inputs, 3 in each section, were applied to introduce the culture medium. Within one section we kept the medium identical while between sections, the concentrations of anthrax toxin were different.

We tested the performance of medium mixing that driven by peristaltic pumps embedded on-chip. Culture media with different toxin concentrations were loaded into the pre-designed sections, respectively. A typical mixing took 10 min, through a 50 Hz pumping action using the integrated 3-valve peristaltic pumps. After mixing the medium was uniformly distributed among all the connected chambers in both the experimental and control groups.

In each device, we used 4 sections to simultaneously carry out the experiments with 3 toxin dosages, and an extra group without toxin as the reference. Cells that died during the experiment would detach from the chamber bottom and were then flushed out of the device with other metabolic wastes through medium mixing and changing. The LM/shRNA157B cells in Group C would not produce sufficient DKK1 while they could continuously receive extracellular DKK1 secreted from the cells in Group B. In conventional practice, we replace the toxin-containing medium with fresh medium after 48 h treatment. However, when cultured on chip, the chamber is only 0.05 mm thick so we have to introduce the fresh medium more frequently to keep the cells healthy. We noticed that medium replacement every 3 to 6 h is necessary to maintain the cell viability at expected cell density in culture chamber. To minimize the DKK1 concentration fluctuation, we chose to replace the medium every 6 h.

We examined the cell viability on-chip through the live/dead staining with Calcein AM (a green fluorescent dye for indicating live cells) and Ethidium Homodimer-1 (EthD-1, a red fluorescent dye for indicating dead cells via nucleic acid labeling). In every section, we obtained the toxin sensitivity of LM and LM/shRNA157B cells in groups A and D, respectively, and collected the cell survival data of groups B and C in response to toxin through the fluorescence microscopic imaging. Fluid flow also makes the fluorescent staining automated and uniform. To test the sensitivity of LM and LM/shRNA157B cells to the toxin, we typically cultured the cells inside the micro-chambers for 48 h with the toxin treatment. As LF does not cause lethality in most cell types except macrophages where the cleavage of MAPKK triggers apoptosis^38^, we employed FP59, a PA-binding fusion protein containing LF amino acids 1–254 and the catalytic domain of Pseudomonas aeruginosa exotoxin A, to investigate the actions of PA^39^,^40^. The conventional culture experiments have shown that the LM/shRNA157B cells, with DKK1 expression knocked-down, exhibit much higher level of resistance to the chimeric toxin than the wild-type LM cells (Figure 3b).

**Figure 4:**
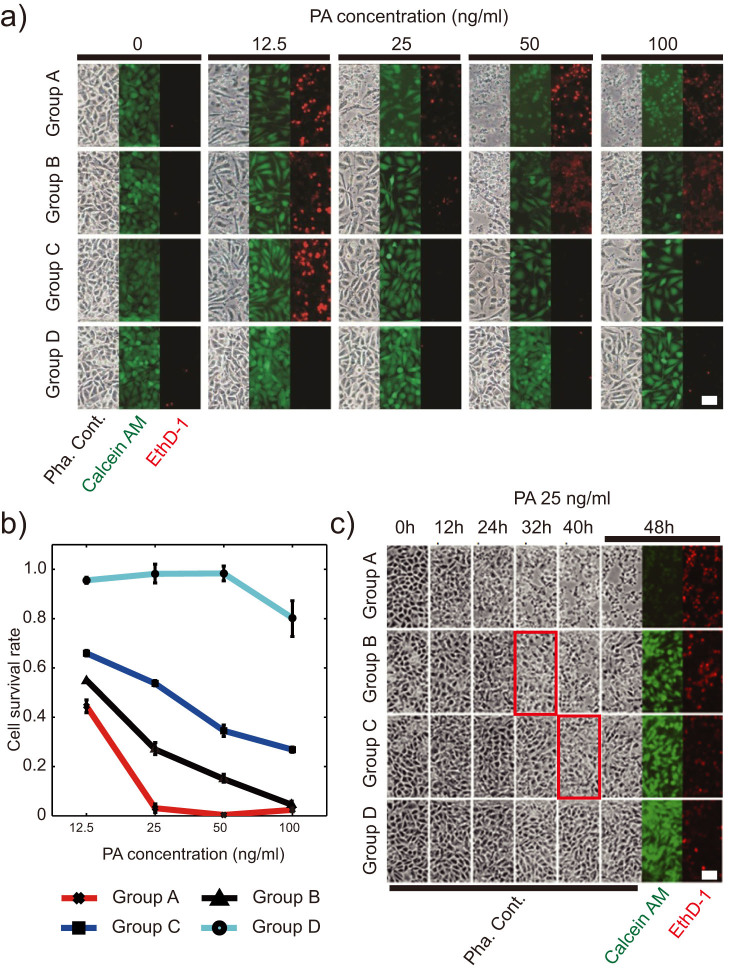
The cytotoxicity assay of microfluidic-cultured cells with DKK1-experssed conditioned medium. (a) Images of cells at the end of toxin assay, with different PA concentration. For each culture chamber, phase contrast image and fluorescence images for Calcein AM and EthD-1 are captured for analysis. (b) Survival rates of cells that treated with PA at different concentrations. (c) Time-lapse microscopic images (phase contrast, and fluorescence for both Calcein AM and EthD-1) at the time points of 12, 24, 32, and 40 h for each chamber. Red boxes highlight the morphological changes associate with the beginning of cell death (50 ng/ml of FP59 is supplied with PA in each assay). Scale bar: 20 μm.

Unlike the conventional assays that typically used MTT assay to monitor the cell viability with limited control of the culture environment, we were also able to use this chip-based method to observe the morphological behaviors of cells and their response to different culture conditions with precise control. Both the wild-type LM cells and LM/shRNA157B cells showed the normal status when they were cultured on-chip without toxin treatment, with survival rates close to 100% (Figure 4a). However, the cell viability in groups A was gradually decreased when we elevated toxin concentration in the culture medium. It is noticeable that in some cases the detachment of dead cells might result in the decrease of cell density. In contrast, LM/shRNA157B cells in group D, in which DKK1 expression was knocked-down, were clearly resistant to toxin when PA concentration did not exceed 50 ng/ml; even 100 ng/ml of PA only slightly brought down the viability to ~80% (Figure 4b). However, the LM/shRNA157B cells cultured in group C, fed with the conditioned medium containing DKK1 secreted from wild-type LM cells cultured in group B, did not hold the resistance to toxin as the cell survival rate obviously decreased upon toxin introduction. This observation clearly suggested that the extracellular DKK1 facilitated the uptake of toxin by cells. In the meantime, the group B with wild-type LM cells that continuously provided DKK1 to cells in group C, showed a significantly improved viability due to the dilution and extra consuming of secreted DKK1. We also observed the higher viability of cells in group C than those in group B, implying that the DKK1 concentration in the medium of group C (receiving end) was lower than that in group B (supply side) in the dynamic DKK1 supplement.

We then monitored the dynamic response of the cells to toxin to fully exploit the advantages of large-scale integrated microfluidic devices. The chip was incubated in the homemade culture chamber on the sample stage of a microscope. The chamber could maintain an environment of the mixture of air and 5% CO_2_ at 37°C without interfering microscopic observation and image acquisition at the same time. We initiated the toxin assay when the cells grew to confluence on-chip. We took time-lapse bright-field microscopic images of each chamber when toxin-containing medium was introduced into the chip, and at the time points of 12 h, 24 h, 32 h, and 40 h. Finally, we stained all the chambers with Calcein AM/EthD-1 at 48 h (Figure 4c).

When we used 25 ng/ml of PA and 50 ng/ml of FP59, wild-type LM cells (group A) exhibited a clear dead trend at ~24 h after toxin was provided, while at that time all the other three groups did not show obvious cell death. Later (~32 h), we found that the cells in group B began to shrink and the shape turned to round. Similar morphological changes had also been observed in LM/shRNA157B cells of in group C, but with a time delay of ~8 h. This time difference was probably due to the difference in DKK1 concentration. Through the real-time observation of the cell status in microfluidic devices, we could obtain the accurate information of cells' dynamic response to toxin, and provide extra evidence in analysis of the biological functions of DKK1 in anthrax lethality.

## Conclusions

In summary, we have presented a novel microfluidic live-cell assay to quantitatively study cell response to toxin using continuously supplied conditioned medium that produced by a different group of cells in real-time. All the cells were cultured on-chip and the secreted factors could be specifically delivered into certain cells through microfluidic fluid manipulation. We applied this assay to study the effect of a secreted protein, DKK1, on cell susceptibility to anthrax toxin in LM/shRNA157B cells, in which the endogenous DKK1 expression had been knocked-down. Highly integrated micro-valves and micro-pumps made the multiplex experiments possible. We have realized 128 parallel culture chambers, as well as 96 medium replacement chambers, to operate simultaneously, with two types of cells, four toxin concentrations, and four replicates. This compact device was fully automated by computer programming with real-time microscopic observation in-situ. Our demonstration has shown that experiments that are conventionally difficult to operate or hard to reproduce, such as the cell culture requiring precisely and dynamically controlled conditioned medium, can be readily performed using this platform, with much less consumption of materials and much higher accuracy and reproducibility.

## Methods

### Conventional cell culture

All cells were cultured in Dulbecco's modified Eagle's medium (DMEM, Invitrogen) supplemented with 10% (v/v) fetal bovine serum (FBS, Invitrogen) and 50 units/mL of penicillin-streptomycin (Invitrogen), and incubated in a humidified incubator containing 5% CO_2_ at 37°C.

### Cell transfection

Lentiviral shRNA expression constructs pLKO.1 carrying shRNA specifically targeting on mouse DKK1 gene were obtained from OpenBiosystems, Inc. DNA transfections were performed with Polyethylenimine (PEI)-mediated method^41^. Lentivirus were produced by transient transfection of HEK293T cells by shRNA expression construct with pCMVΔR8.74 and pMD2.G-VSVG. Stable shRNA expression LM cells were achieved through viral infection followed by puromycin (2 μg/mL) selection. Real-time PCR was performed to confirm that mDkk1 expression was decreased in LM cells.

### Off-chip cytotoxicity assay

PA were produced using plasmid pET-22b-PA^42^ and FP59, a surrogate of LF consisting of the N-terminal 1–254 residues of LF fused to the catalytic domain of Pseudomonas aeruginosa exotoxin A, was purchased from List Biological Laboratories, Inc. Cytotoxicity assays were performed as described^8^ with MTT (AMRESCO). Each data point and related error bar shown in the figure for the MTT assay represent the average results from six replicates.

### Real-time PCR

RNA of cultured cells was isolated using EasyPure RNA kit (Transgen, ER101-01), and the cDNAs were synthesized by PrimeScript 1st Strand cDNA Synthesis kit (TAKARA, 6110A). Real-time PCR was performed with Brilliant III Ultra Fast SYBR Green QPCR Master Mix (Stratagene, 600882) on Stratagene Mx3005P qPCR system. β-actin transcript levels were measured as internal controls.

### Sequences

Mouse DKK1 target of lentiviral shRNA: AGAGCCATCATTGTAAACACGGCTTTTTG. PCR primers for mDkk1: 5′-ATGAGGGCGGGAACAAGTAC-3′, 5′-GAGCCTTCTTGTCCTTTGGTGT-3′. PCR primers for mouse β-actin: 5′-CCAGCCTTCCTTCTTGGGTAT-3′, 5′-GTAACAGTCCGCCTAGAAGCA-3′.

### Fabrication of multilayer microfluidic devices

All devices were fabricated using multilayer soft lithography^43^. Devices are composed of three layers of polydimethylsiloxane (PDMS, RTV615 kit, GE), bonded to a cleaned glass slide (7 × 10 cm). Two separate master molds, one for the fluidic layer and the other for the control layer, were fabricated by photolithography. The silicon wafers were treated with hexamethyldisilazane (HMDS, Alfa Aesar, USA) vapor for 3 min at 25°C before being coated with photoresist. The hybrid master mold of the fluid layer was fabricated through a multi-step photoligraphy to form the molds with different thickness. The flow channels were fabricated by double spin-coating positive photoresist (P4620, AZ Electronic Materials) to a thickness of 25 μm. After photolithographic etching, the patterned positive photoresist was re-flowed on a hot plate ramped from 30°C to 220°C at 6°C/h, to obtain rounded channel profiles, with a peak height of about 25 μm. Subsequently, the chambers for connection were patterned by 15 μm thick negative photoresist (SU-8 2025 MicroChem, Newton, MA). Finally the hybrid master mold was baked at 160°C for 1 h to fully crosslink the SU8. The mold of the control layer had 15 μm thick features made by P4620 positive photoresist (AZ Electronic Materials). Before the fabrication of PDMS chips, both molds were treated with trimethylchlorosilane (TMCS, Sinopharm, China) vapor for 5 min at 25°C. The fluidic layer was made by pouring PDMS (5:1, elastomer to crosslinker ratio) onto its mold to a thickness of 5 to 6 mm. The control layer of the chip was made by spin-coating PDMS (20:1, elastomer to crosslinker ratio) onto the mold at 1400 rpm for 60 s. Then the fluid and control layers were baked at 80°C for 20 min and 30 min, respectively. After the fluidic layer was peeled off from its mold and hole-punched, it was aligned over the control layer, and then bonded at 80°C for 45 min. The bonded layers were peeled off from the control mold, hole-punched, then placed on a glass slide with a thin, cured PDMS layer (10:1, elastomer to crosslinker ratio). Finally, the whole chip was incubated at 80°C for at least 6 h. The complete chip is shown in Figure S1.

### Automation

All the valves in the chip were driven by computer controlled solenoid valves. When the process of medium exchange was confirmed, the procedure was performed automatically using a designed LabVIEW (National Instruments, Austin, TX, USA) program. All imaging instruments were controlled by our own program script written in MATLAB (MathWorks, Natick, MA, USA).

### Microfluidic cell culture

At the beginning of each experiment, Pluronic F-127 (Sigma-Aldrich; 0.2% w/w in PBS, filter-sterilized) was incubated for 1 h inside the entire flow channels. This passivated the PDMS surfaces and therefore prevented the nonspecific adsorption of DKK1 or anthrax toxin molecules, and further eliminate the possible transportation loss of such molecules^34^. Before loading the cells on-chip, a sterile solution of fibronectin (Invitrogen, 100 μg/mL in PBS) was incubated in the culture chambers for at least 1 h and then rinsed with growth medium. The cells were trypsinized to single cells, centrifuged, and re-suspended. Then, the cells were loaded through the inlets on chip with tygon tubing. When cells became confluent, the fresh culture medium (10% FBS, 50 units/mL of penicillin-streptomycin in DMEM) were applied every 4 h. The chip was incubated in the homemade device which contained two indium−tin-oxide (ITO) glass plates on both bottom and top sides of the chip, and the temperature of the two plates were controlled by two PID controllers, and a tube applied 5% CO_2_, in order to maintain an environment of the mixture of air and 5% CO_2_ at 37°C. The cells were incubated in the chip for 8 hours before toxin assay.

### Microfluidic toxin assay

The chip was divided into four parts, to culture cells under three kinds of toxin conditions, including PA of 12.5 ng/ml, 25 ng/ml, 50 ng/ml, plus FP59 (50 ng/mL), and control medium without toxin respectively, as shown in Figure 2a in different colors. The cell inputs C1 and C3 are delivered the wild type cells and the C2 and C4 are delivered the mutation type 157 shRNA cells. Before toxin assay, the cells were incubated in the chip for 8 hours. Three concentrations of toxin culture medium and fresh control medium are delivered into 4 inlets respectively. The peristaltic pumps composed of in series cascading valves, located between the pair of cell culture and medium chambers, allow the circulation of the condition medium around. By changing the configuration of the valves, cells in each section were made in 4 different culture environment, including wild type cell which culture medium only switch with medium chamber, wild type cell which culture medium switch with mutation cell through the medium chamber in the middle, mutation cell which were supplied of conditional medium contains DKK1 from the wild type cell, and mutation cell which culture medium only switch with medium chamber. The velocity of the conditional medium mixing is controlled by the number of actuation cycles applied to the pump valves, and the flow speed by the frequency at which the valves are switched. Typically, the time of medium turning around a cycle is about 60 s.

### Cell viability staining

All assays are performed after toxin added 48 h in culture in the device. Cell viability is determined by staining with two-probe solution which contains 2 mM Calcein AM (Invitrogen) and 4 mM ethidium homodimer-1 (EthD-1, Invitrogen) in PBS. The stain solution is incubated for 30 min before imaging.

### Image taken and analysis

Phase-contrast and fluorescence images of cells viability in each chamber were recorded by an automated microscope (TE2000-E, Nikon) with a CCD camera (Olympus, DP72). Time-lapse images of cell growth and dead curve were obtained at the time point 0, 12 h, 24 h, 32 h, 40 h and 48 h after toxin added. Home-developed MATLAB scripts were employed to perform image analysis and data statistical analysis. We developed our own MATLAB script to recognize and calculate the cell survival rate from fluorescent images. We picked up the red channel from the images labeled with EthD-1, and turned grey scale image to binary image, then summed total red pixels, representing dead cell numbers. The total green pixels were calculated according to the same method, representing live cell numbers. Since the enzyme was not completely inactivated, some dead cells were marked with green and red at the same time, so we defined the double labeled cells were dead cells, that statistical significance of the living cells is green pixels minus double labeled pixels. Because lots of dead cells were off the base, and washed away when the culture medium updated, so we considered the live cell numbers in the control group as a starting cell numbers in experimental group. The survival rate was calculated by dividing the number of the living cells in experimental group by that in control group.

## Author Contributions

Y.H. and W.W. conceived and designed the methods and experiments. J.S., C.C., Z.Y., Y.P., Y.Z. and L.Q. conducted the experiments and data analysis. J.S., W.W. and Y.H. wrote the manuscript.

## Supplementary Material

Supplementary Information Supplementary Information
