# Supplementary material for: A microfluidic live cell assay to study anthrax toxin induced cell lethality assisted by conditioned medium
Source: Sci Rep. 2015 Mar 3;5:8651. doi: 10.1038/srep08651 (PMC4346806; doi:10.1038/srep08651)
Supplement: Supplementary Information [file srep08651-s1.pdf]

Electronic Supplementary Information

A microfluidic live cell assay to study anthrax toxin induced cell lethality assisted by conditioned medium

Jie Shen,[1,2,3] Changzu Cai,[3] Zhilong Yu,[1,2] Yuhong Pang,[1,3] Ying Zhou,[1,2] Lili Qian,[3] Wensheng Wei,[*,3] and Yanyi Huang[*,1,2]

1 Biodynamic Optical Imaging Center (BIOPIC), Peking University, Beijing 100871 (China)

2 College of Engineering, Peking University, Beijing 100871 (China)

3 School of Life Sciences, Peking University, Beijing 100871 (China)

E-mail: yanyi@pku.edu.cn, wswei@pku.edu.cn

Supporting figures

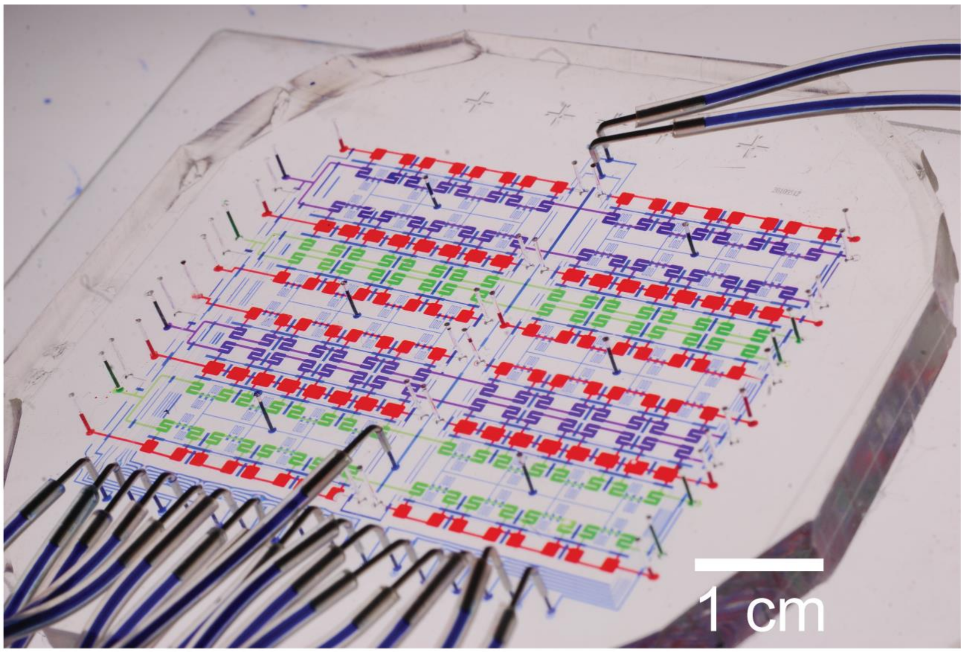

Figure S1. Microphotograph of the microfluidic device. The micro-channels are filled with dyes to indicate the multilayer structure and the different functional components.

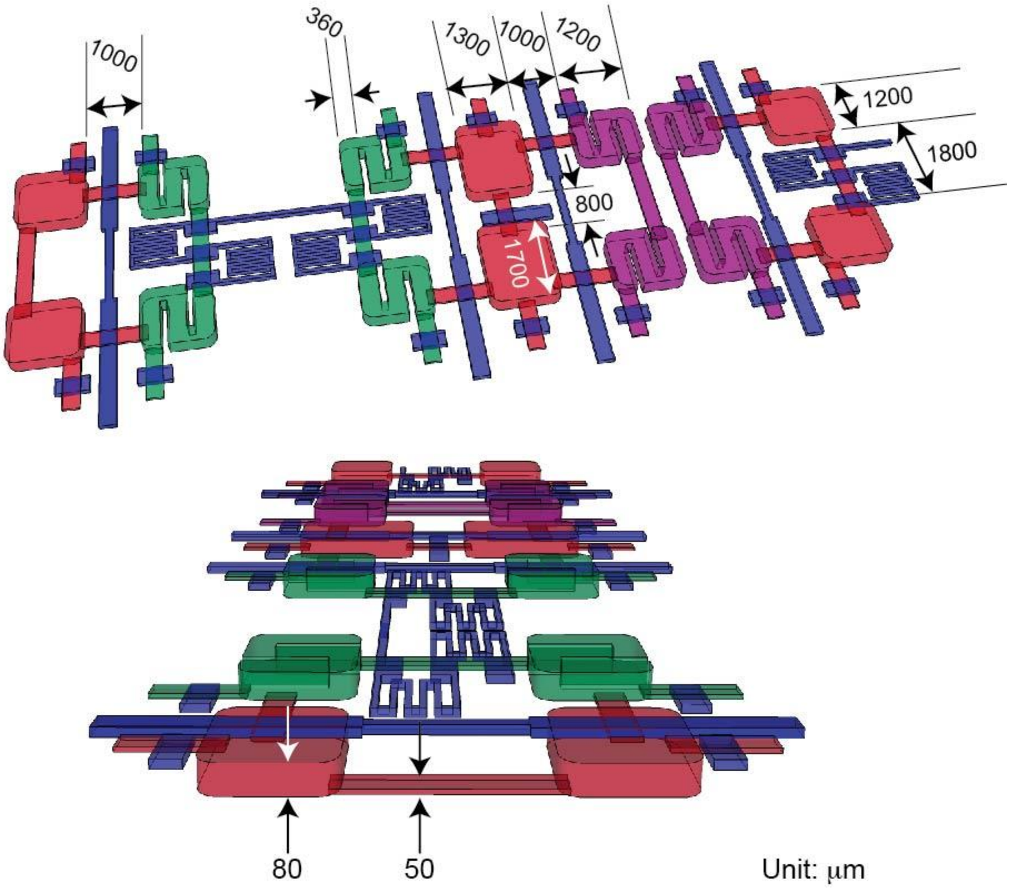

Figure S2. The dimensions of critical components of the microfluidic device.

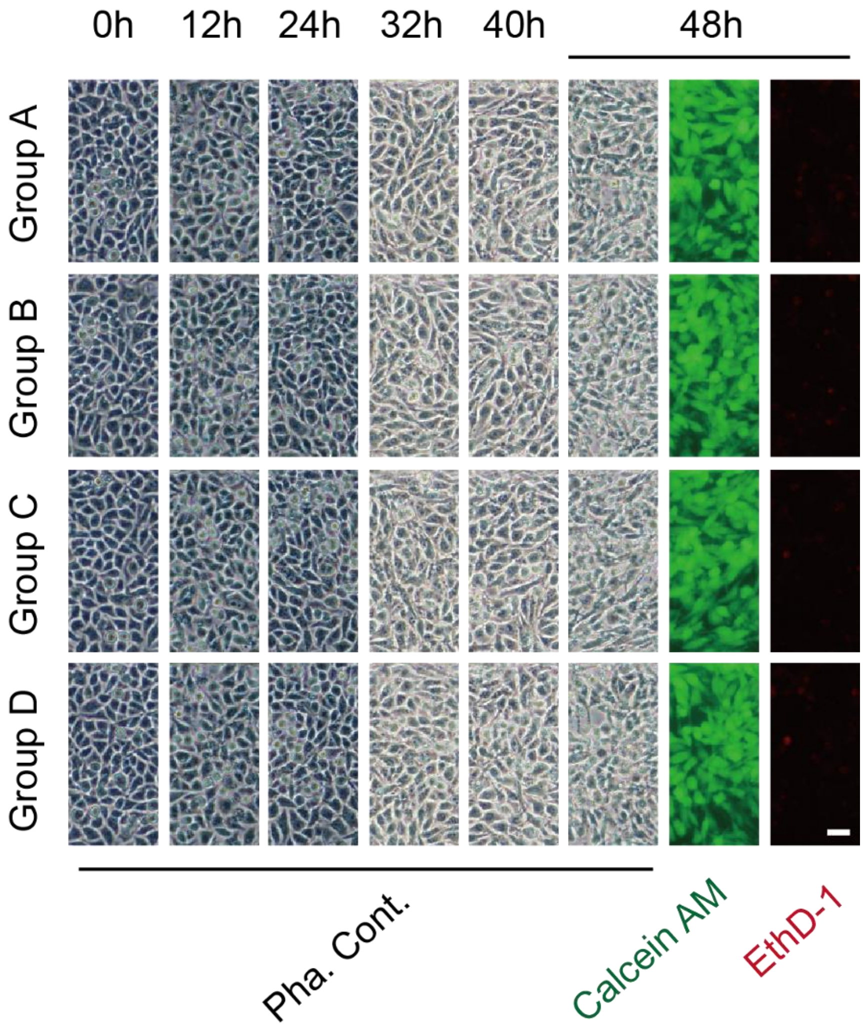

Figure S3. The time-lapse imaging of the control experiment in the on-chip toxin assay. The phase contrast microscopic images of each chamber were taken at the time points of 12 h, 24 h, 32 h, and 40 h, and 48 h. Then all the chambers were stained with Calcein AM/EthD-1. Scale bar: 20 μm.

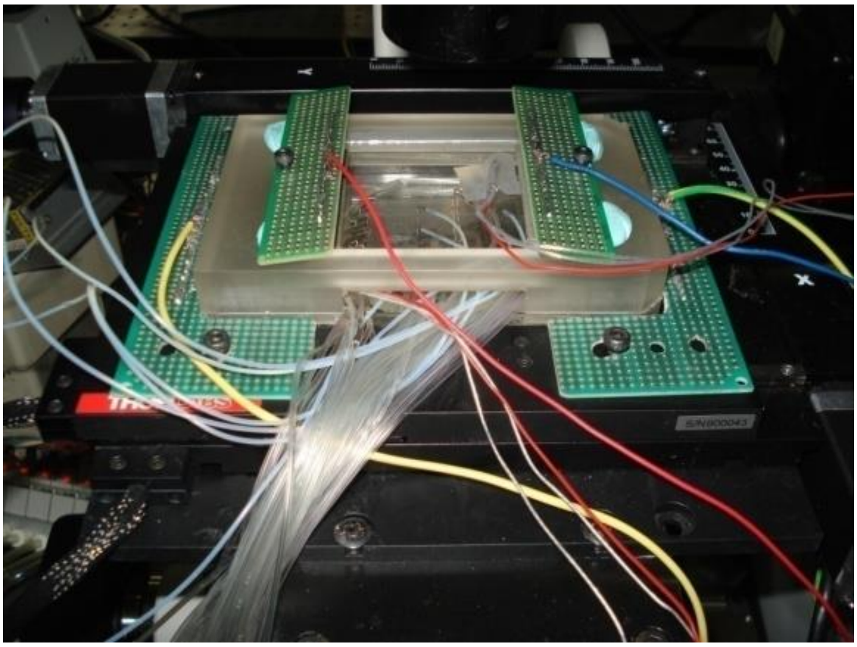

Figure S4. A home-made live-cell incubation setup for long-term microfluidic cell culture on top of a microscope stage. The dynamic response of the cells to toxin was monitored under the microscope. The chip was sitting inside the incubation chamber. The chamber could maintain an environment of the mixture of air and 5% CO_2 at 37 °C without interfering microscopic observation and image acquisition at the same time.

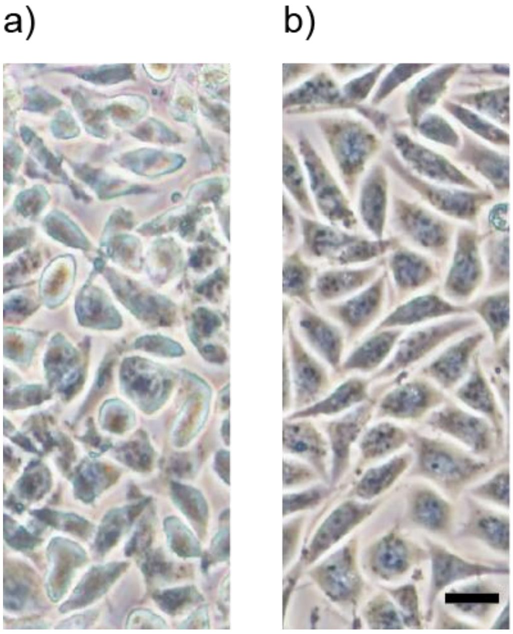

Figure S5. The phase contrast microscopic images of LM cells that been cultured on-chip for 24 h. LM cells are sensitive to cytokines in the conditioned media and to the shear stress during the medium refreshment. (a) If the cell culture chamber was completely replaced by fresh medium every 4 h, the cell started to detached from the surface and eventually died in the chambers. (b) With the design that containing chamber 3 and 4 for medium replacement (see Figure 2c in the main text), the medium change did not flush out all conditioned medium, providing a suitable condition for healthy culture of cells. Scale bar: 10 μm.

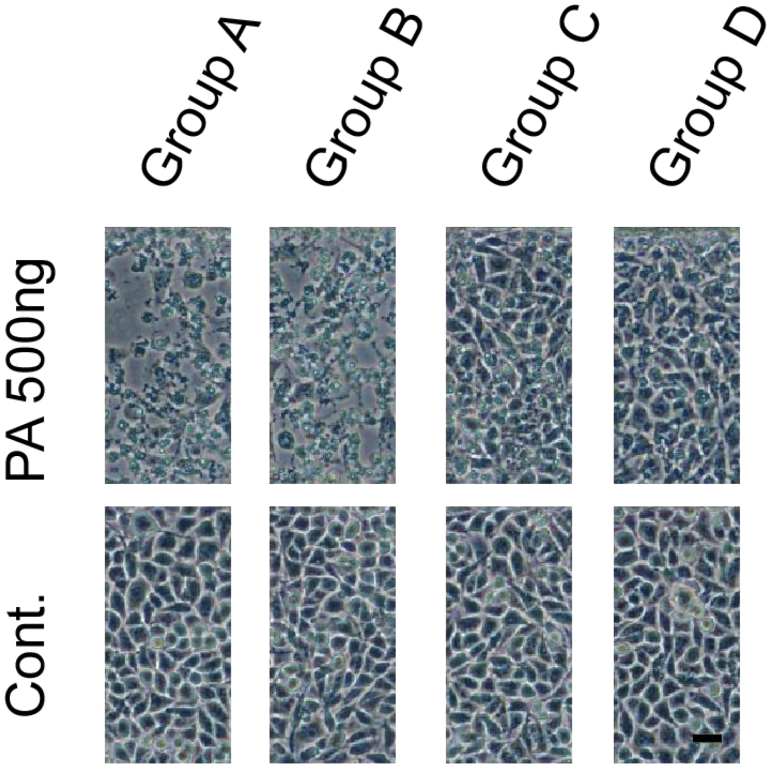

Figure S6. The phase contrast microscopic images of cells cultured with high concentration of toxin (PA: 500 ng/ml, FP59: 50ng/ml). Images were taken after 12 h of culture. Wild-type LM cells (group B) exhibited a clear dead trend at 12 h after toxin was provided, while LM/shRNA157B cells (group C) at that time did not show obvious cell death. Later (~48 h), LM/shRNA157B cells (group C) began to shrink and the shape turned to round, similar to LM/shRNA157B cells in group D. Because wild-type LM cells (group B) quickly dead under the treatment with high concentration of toxin, the LM/shRNA157B cells (group C) did not receive sufficient DKK1, still holding the resistance to toxin. Scale bar: 20 μm.
